# Canadian perspectives on loneliness; digital communication as meaningful connection

**DOI:** 10.3389/fpubh.2024.1389099

**Published:** 2024-10-22

**Authors:** Lauren Dwyer

**Affiliations:** Department of Communication Studies, Faculty of Business and Communication Studies, Information Design, Mount Royal University, Calgary, AB, Canada

**Keywords:** loneliness, communication studies, COVID-19, digital technology, public health, Canadian mental health policy, mental health

## Abstract

This perspective piece considers loneliness and its relationship to communication, connection, and technology by reviewing the origins and lessons from the field. It begins with a search for an operational definition, then examines the differences between experiential (situational/isolation-based) and existential (continuous, non-situational) loneliness. Technology is addressed as both a hindrance and a tool for alleviating loneliness with the example of companion robots as an emerging technology for loneliness mitigation. Cultural differences in experiences of loneliness, specifically as a public health issue, are in the context of the COVID-19 pandemic in Canada. Concepts of social and emotional loneliness, individualism and collectivism, socioeconomic status, vulnerability, and lived experience are explored and provide an emphasis on ‘meaningful connection’ in the study of loneliness.

## 1 Introduction

Loneliness is a complex, non-clinical condition with distinct mental and physical implications, including depression and cardiovascular issues that, if left unaddressed, can even affect mortality rates (1, 2). Research in this field is unique; loneliness is not a mental illness that can be diagnosed yet it plays an identifiable role in the experiences of individuals diagnosed with depression (2). Public perception of loneliness has evolved, especially with the advent of digital connectivity and social media’s influence on individual loneliness levels (3, 4). Nevertheless, it remains an experience that has been, at least until recent developments with isolation and COVID- 19, considered universally shameful; to be lonely is to be a social outcast, to under-perform at something inherently human - that being a connection to other humans (4, 5). The historical context shapes the understanding of loneliness, highlighting the subjective nature of its experience and its potential to trigger personal growth (6).

On a global scale, the WHO’s Commission on Social Connection (2024–2026) underscores the pervasive nature of loneliness and social isolation, emphasizing its serious impacts on physical and mental health across all ages and regions, and advocating for it to be recognized as a global public health priority. Additionally, organizations such as the Global Initiative on Loneliness and Connection, represent international efforts to address loneliness through collaborative public health campaigns and initiatives across 11 partner countries and organizations.

This article presents the author’s viewpoint of communication as meaningful connection as a strategy to address the issue of loneliness, particularly with insights from a Canadian perspective. It explores loneliness, differentiating between experiential and existential loneliness. Technology is considered for its dual capacity to exacerbate loneliness as well as serve as a remedy, especially within a Western framework. The narrative extends to cultural variations in perceiving loneliness as a public health concern and its manifestation during the COVID-19 pandemic. The exploration includes themes such as social and emotional loneliness, the dichotomy of individualism versus collectivism, the impact of socioeconomic status, vulnerability, and personal experiences. Through this examination, the concept of communication as ‘meaningful connection’ provides a pivotal lens through which to understand and address loneliness.

## 2 Defining loneliness

Bound Alberti (7) emphasizes loneliness as a complex emotional cluster influenced by a variety of factors rather than a singular emotion. The physical, psychological, and situational facets of loneliness affect overall health and are intricately linked with social support (8, 9). High stress associated with loneliness can worsen health outcomes, including during the COVID-19 pandemic (10–12). Despite the stigma diminishing over time, loneliness research has had to find creative methodologies due to the reluctance to admit such feelings (13).

The stigma surrounding this experience requires that some researchers seek unorthodox solutions for evaluation, as asking individuals if they have experienced loneliness can lead to inaccurate and roundabout responses (13). Rokach (13) notes in his work that “No one, in my 30 years of researching this topic, has ever had the courage to admit, in public, that he or she is lonely” (p. 1). General public acceptance of mental health and de-stigmatization campaigns may have caused shifts in the last 10 years; however, loneliness is still somewhat stigmatized as a need to be perceived as belonging to the social groups we interact with (13). Loneliness is also addressed in the literature as akin to hunger, a force that generates a desire for the individual to seek what they are lacking rather than a negative experience that offers no potential positive outcomes (6, 14).

Viewing loneliness through a phenomenological lens offers a subjective perspective, enriching the understanding of its impact (15, 16). Loneliness, while related to isolation at times, is not dependent on being physically alone for the experience to be felt (6). Individual perceptions of social experiences have major implications for people living with loneliness’s physical health and well-being. This emphasis on perception, however, causes difficulties for those who seek to find a universal definition of the experience.

For the purposes of this perspective, loneliness is understood as an experience within the context of complex emotion, dependent upon physical and psychological circumstances. The concept of loneliness as experiential rather than circumstantial and a complex compendium rather than as a single emotional experience allows researchers to consider the external social factors that contribute to loneliness (7, 17). This operational definition depicts loneliness as an experience of a lack or loss of meaningful connection. By breaking down this definition, key terms arise. In this context, “perceived” is used as in each of the previously mentioned articles, researchers note the importance of individual perception on the experience of loneliness. The terms “lack or loss” differentiate and give value to both existential (general non-situational lack) and experiential (distinct event-driven loss) loneliness. The term “meaningful” distinguishes shallow and deep social interactions, as Hawkley and Cacioppo (14) stress the value of quality over quantity of interactions. Finally, “connection” encompasses both social interactions and a feeling of connectedness and belonging that is not always dependent on the volume of communication.

## 3 Cultural considerations

Exploring loneliness through the lens of communication studies illuminates how interpersonal communication patterns and media usage differ across cultures, impacting feelings of loneliness. Cultural comparisons reveal that loneliness varies between individualistic cultures, which emphasize personal achievement, and collectivist cultures, which value group cohesion (18, 19). Research indicates that individualism tends to correlate with increased loneliness, especially among younger men (18). In contrast, collectivist cultures, which promote group belonging, generally report lower loneliness levels due to higher social integration (19) (p. 791). Cultural heritage also influences loneliness perceptions, with those from North American individualistic backgrounds reporting greater loneliness compared to those from collectivist backgrounds (5). Van Staden and Coetzee (20) emphasize that cultural conceptions of loneliness involve expectations of empathy and social closeness within relationships.

Globally, loneliness is linked to cultural factors such as emotional distress and social disconnection, with North Americans typically experiencing higher levels (5). However, the aspiration to overcome loneliness is a common thread across cultures (21). For instance, older adults in Sweden and Hong Kong express a shared theme of “overcoming” existential loneliness, signifying the universal nature of the desire for connection (21).

## 4 Evaluating metrics

### 4.1 Metrics for identifying loneliness

The UCLA Loneliness Measurement Tool, developed by Russell and colleagues in 1978 and revised in 1996 (version 3), is the most commonly used measure of loneliness in general populations (22). Originally a 20-item scale designed to assess subjective loneliness and isolation, it has been modified to a simpler 3-item scale for telehealth surveys (23). The tool focuses on subjective experiences of social companionship, asking participants about feelings of companionship, being “left out,” and isolation, allowing researchers to identify the root causes of loneliness (22). While the scale’s brevity and clear language are advantages, its ability to pinpoint the exact cause of loneliness is limited.

The De Jong Gierveld Loneliness Scale, available in 6 or 11-item versions, is another popular tool for measuring loneliness (24). It differentiates between social loneliness (lack of a broad social network) and emotional loneliness (lack of intimate relationships). The scale’s use of both positive and negative language helps prevent automatic responses and encourages deeper participant reflection. However, the longer version can be cumbersome to use in surveys, leading to the development of a shorter 6-item scale.

The Campaign to End Loneliness Measurement Tool, co-designed by researchers, professionals, and older adult individuals, serves as a marker for evaluating interventions over time (22). It assesses contentedness with friendships and relationships, comfort in asking for help, and relationship satisfaction. Like the UCLA and De Jong scales, this tool emphasizes the importance of connection and perception of relationship strength. These scales share a focus on subjective experiences and meaningful relationships, aligning with the definition of loneliness as a lack or loss of meaningful connection.

The COPE scale, developed by Carver (25), uses a simplified version of previous loneliness scales and focuses on the coping strategies participants use to address their loneliness. Unlike other scales, COPE is designed to assess coping methods both retroactively and in real-time. This makes it ideal for studying loneliness in populations with significant isolation experiences, such as astronauts or workers in remote locations. The COPE scale includes categories similar to those proposed by Rokach and Brock (26), such as reflection/acceptance, self-development/understanding, social support network, distancing/denial, religion/faith, and increased activity, offering insights into common loneliness management strategies.

### 4.2 Individual management

To examine loneliness through a communication studies lens we first look to the cognitive discrepancy model of loneliness by Perlman and Peplau (27). This theory emphasizes perceived social involvement versus desired levels of social involvement, which underscores the centrality of communication in experiencing and evaluating loneliness. This model of loneliness suggests that loneliness is experienced when an individual’s perceived social involvement does not live up to their desired levels (28). By examining loneliness through a communication lens, we can better appreciate how discrepancies in expected and actual social interactions contribute to feelings of loneliness, highlighting the importance of effective interpersonal communication.

The mitigation of experienced loneliness is not necessarily considered treatment, as loneliness is not specified as a disorder or condition by the DSM-5-TR™.^1^ Therefore, actions taken by lonely individuals to alleviate their negative experiences as “treatment” but rather as coping or management strategies for their existing negative experiences. Deckx et al. (29) explore various coping strategies, including reflection/acceptance, self-development/understanding, social support network, distancing/ denial, religion/faith, and finally, increased activity. Quality and quantity of social support are noted as important factors in how individuals experience loneliness and may hold the key to management (30).

Expectation management regarding personal relationships plays a role in coping, specifically by lowering relationship expectations or by improving current relationships to meet expectations (31). Quality and quantity of social support are noted as important factors in how individuals experience loneliness and may hold the key to management (30). The studies also note that the type of loneliness experienced (emotional vs. social) plays an important role in how one goes about coping and the effectiveness of the strategy (29). Emotional loneliness is defined by Cacioppo et al. (1) as “the perceived absence of a significant someone (e.g., a spouse), that is, a person one can rely on for emotional support during crises, who provides mutual assistance, and who affirms one’s value as a person” while social loneliness is defined as “the perceived presence/absence of quality friendships or family connections, that is, connections from the ‘sympathy group.’” For example, those whose loneliness stems from an absence of emotional support will require different coping techniques than those who require larger group settings or loser connections to mitigate their loneliness. Each strategy involves individuals in some way modifying their behavior, either through avoidance or seeking of sociality or through self-reflection and modification of expectations. The strategies that individuals choose to employ to cope with their loneliness are integral to determining how technology can assist in facilitating these strategies. Communication studies provide a unique framework for understanding loneliness, suggesting that interventions aimed at improving communication skills and enhancing social connections could be effective in addressing this complex issue.

## 5 Discussion: Canadian public health and the COVID-19 context

Loneliness, while extensively researched, has been predominantly focused on special populations like the older adult or disabled, neglecting its broader impact (32). Various factors influence how loneliness affects mental health, especially during the pandemic, with certain demographics reporting deteriorating mental health conditions (33–40). Furthermore, food insecurity has been linked to poorer mental health outcomes (38). Canadian youth, in particular, have experienced a significant decline in mental health, exacerbated by COVID-19, with a notable decrease in those reporting excellent mental health and an increase in negative mental health impacts, especially compared to older populations (39).

The Mental Health Commission of Canada (MHCC) promotes a recovery-oriented approach to managing mental illness, emphasizing a journey toward a fulfilling life and the expectation of recovery (41). This approach, which encompasses personal responsibility and a return to the workforce, is linked to the management of loneliness and is expected to be adopted by the Canadian government (41). The moralization of health, as discussed by Cederström and Spicer (42), aligns with the MHCC’s philosophy and highlights the stigma related to social connection, further complicated by the paradox of social media use, which can increase loneliness (3).

The COVID-19 pandemic has spotlighted the prevalence of loneliness in Canada, with reports of over half the population struggling due to social distancing, although technology has been seen as a mitigating factor (43). Mood disorders, often comorbid with loneliness, affect 11.6% of Canadians, with access to mental health services being limited by long wait times or high costs of private care (39). Similar trends have been observed in other Western countries; a survey by the Campaign to End Loneliness in the United Kingdom found that 45% of adults feel occasionally, sometimes, or often lonely. One group that has led the Canadian landscape in loneliness initiatives is GenWell, an organization dedicated to the social health of Canadians (44). Founded with the mission to enhance individual well-being and societal health, GenWell Project advocates for intentional, face-to-face interactions as a remedy to the growing epidemic of loneliness. GenWell’s primary focus is on social connection and the mitigation of loneliness unlike other Canadian mental health organizations such as the Canadian Mental Health Association (CMHA) and the Center for Addiction and Mental Health (CAMH) focus primarily on mental health and addiction as a whole, with only some initiatives for isolation and loneliness. Genwell faces challenges, however, as tools for communication can act as both facilitator for connection and social isolator.

The role of digital communication is crucial in these initiatives. In Canada, digital tools have been leveraged to maintain social connections during periods of physical distancing. Telehealth services, virtual meet-ups, and online support groups have become vital in mitigating loneliness. This mirrors efforts in other Western countries, where digital platforms have been used to create virtual communities and provide mental health support. However, the challenge remains to ensure these digital interactions are meaningful and do not replace but rather complement face-to-face connections. Addressing loneliness effectively requires a balanced approach that integrates both digital and in-person strategies tailored to the specific cultural and social contexts of each country.

## 6 Technology as problem and solution

The role of technology in addressing social connection and loneliness presents a duality of potential problem and solution. At its core, technology facilitates communication and interaction, acting as a bridge for those experiencing loneliness to connect with others. Platforms such as social media allow individuals to maintain social networks, potentially alleviating feelings of isolation (45). However, the quality of these interactions often comes into question, with concerns about superficial connections and the exacerbation of loneliness through dependence on virtual rather than physical interactions (46, 47).

Emerging technologies, particularly social companion robots, offer a promising solution to mitigate loneliness by providing companionship and interactive experiences. These robots are designed to engage users in meaningful interactions, thereby filling the emotional and social void that contributes to loneliness (48, 49). By fostering a connection “with” and “through” these robots, individuals can experience a form of companionship that, while artificial, may offer real emotional benefits. This aligns with Zeller’s (50) human-machine communication (HMC) model, which emphasizes the importance of user experiences and the socio-cultural dimensions influencing communication processes.

The design and implementation of social companion robots must be grounded in human-centered design principles to ensure meaningful connections are established. Zeller (50) model stresses iterative design enriched by user feedback, highlighting the need to personalize interactions to meet the diverse needs of users. For example, addressing communication barriers for individuals with disabilities or considering the digital divide impacting socioeconomic status are crucial for developing effective social robots (51, 52). By integrating these considerations, designers can create robots that not only interact with users but also respond to their unique socio-cultural contexts, thereby enhancing the potential for meaningful engagement.

Companion robots, as part of a broader technological approach, illustrate the nuanced role of technology in managing loneliness. While these robots can offer substantial benefits, there are inherent risks, such as creating dependencies or minimizing human-human connections (53). Future research and design efforts must balance these potential drawbacks with the benefits, striving to enhance the quality of interactions and ensuring technology serves as a tool for genuine social connection rather than a substitute for human presence. By maintaining a focus on human-centered design, technology can evolve to better address the complex emotional and social needs associated with loneliness.

## 7 Conclusion

Loneliness, as explored through the lens of communication studies, reveals the profound impact of meaningful connections on human well-being. This perspective piece has highlighted the dual role of technology as both a facilitator and a barrier to genuine social interactions. The COVID-19 pandemic has further underscored the need for strategies that foster meaningful connections, especially in Canadian society. Moving forward, it is crucial to balance the use of digital tools with initiatives that promote face-to-face interactions and community building. By integrating human-centered design principles in technological solutions and prioritizing quality over quantity in social exchanges, we can address the complex issue of loneliness more effectively. Ultimately, this holistic approach can lead to a more connected and mentally healthy society, where communication serves as the cornerstone of meaningful human relationships.

## Footnotes

^1^The Diagnostic and Statistical Manual of Mental Disorders, Fifth Edition, Text Revision (DSM-5-TR™) is the most recent text at the time of review used by psychologists to analyze, measure, and diagnose mental illnesses.
